# Quaternary structure analysis of IRE1

**DOI:** 10.17912/micropub.biology.000763

**Published:** 2023-03-28

**Authors:** Samirul Bashir, Debnath Pal, Ozaira Qadri, Mariam Banday, Khalid Fazili

**Affiliations:** 1 University of Kashmir, Srinagar, Jammu and Kashmir, India; 2 Indian Institute of Science Bangalore, Bengaluru, Karnataka, India

## Abstract

IRE1 belongs to a type I transmembrane protein family harboring two functional domains, cytoplasmic domain with kinase and RNAse catalytic activity, and the luminal domain, which is involved in the sensing of unfolded proteins. IRE1 molecule undergoes dimerization in the lumenal domain, which functionally activates the catalytic C-terminal domain. IRE1 activation is directly related to transition between monomeric and dimeric forms. We have deduced two quaternary structures from the published crystal structure of IRE1. One structure with a large stable interface that requires large activation and deactivation energy to active IRE1. The other quaternary structure has low dissociation energy and is more suitable for IRE1 oligomeric transition.

**Figure 1. Quaternary structural analysis of IRE1:**
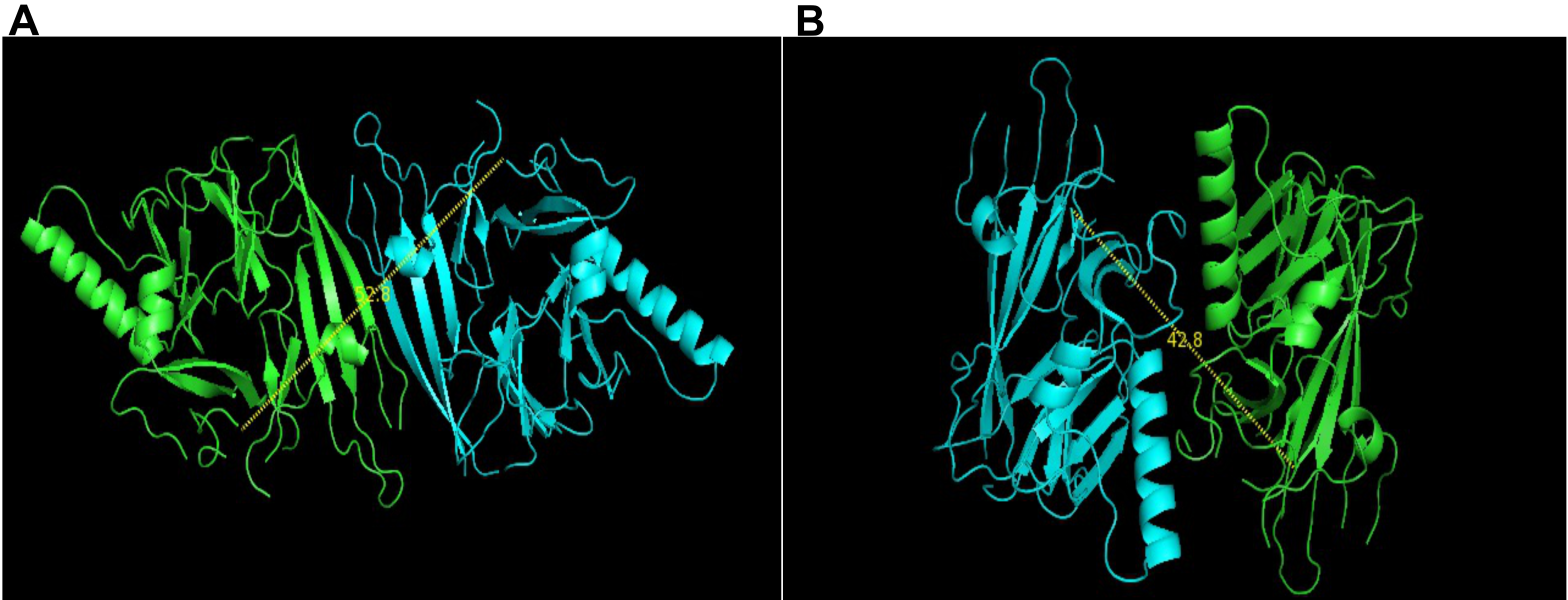
**A.** Quaternary structure form of lumenal domain of IRE1 (PDB ID: 2HZ6). The more stable form of quaternary structure of IRE1 with 62.8 Å (wrongly labelled as 52.8 Å) end-to-end distance between C-termini of monomers. Distance between the C-terminal end are depicted with a dashed line. **B** . Quaternary structure form of lumenal domain of IRE1 (PDB ID: 2HZ6) showing alternative arrangement of a dimer. Less stable form with 42.8 Å end-to-end distance between C-termini of monomers. Distance between the C-terminal end are depicted with a dashed line.

## Description

IRE1 is a sensor protein found in the endoplasmic reticulum (ER) of eukaryotic cells that is involved in the Unfolded Protein Response (UPR), a cellular stress response pathway. It is activated in response to the accumulation of unfolded proteins in the ER, and its activation leads to a cascade of events that help cells cope with the stress (Walter *et al.,* 2011). IRE1 structurally constitutes the sensory N-terminal domain towards the ER lumen, the C-terminal catalytic domain towards the cytosol and a linker region connecting the two domains. IRE1 undergoes dimerization/oligomerization in the lumenal domain, which functionally activates the catalytic C-terminal domain (Bashir *et al.,* 2021). A quaternary structure of IRE1 lumenal dimer was deduced from the crystal structure published earlier (Jiahai *et al* ., 2006) (Figure 1A). The proposed quaternary structure with a large stable interface is formed through a beta-sheet spreading across the dimer subunits. Several mutation studies for residues 105 (Jiahai *et al* ., 2006), 109 (Liu *et al* ., 2003), 123 (Jiahai *et al* ., 2006), 125 (Jiahai *et al* ., 2006) corroborate the effect on dimer formation. There are, however, two problems with this form of the quaternary structure: (i), presence of a strong interface, which implies that a large activation and deactivation energy may be needed to alter the monomer-dimer equilibrium, which may not be adequate for a sensor protein. (ii), the end-to-end distance of the C-termini of lumenal domain between two monomers is 62.8 Å. For the lumenal domain to tether to the cytoplasmic domain across the membrane, a similar N-terminal end-to-end separation for cytoplasmic dimeric form of the protein is desirable. This can be found in the head-to-tail form of the dimeric proteins as shown in (Bashir *et al.,* 2023), where a 65.7 Å separates the dimer, which matches closely with the separation of C-terminal distance in the lumenal domain (Figure 1A). Also, there is a ~193 residue linker region that attach the lumenal domain to the cytoplasmic domain with a 20 residue transmembrane segment located at positions 444-464. This means that there are roughly 76 and 98 residues on the lumenal and the cytoplasmic side that tethers the C-terminal domains, which must also dimerize to perform its biological functions. However, unless the 98-residue segment can form a stable structure, the lumenal---C-terminal domain tether is likely to remain floppy because of the large spatial gap between the two tethers.

The structural perspective from the crystal structure data somehow does not fit well with the fragmented experimental data available, and therefore better strategies need to be developed to synchronize the data. In order to solve this structural discrepancy, here we are presenting an alternative quaternary structural model for dimerization of IRE1 lumenal domain (Figure 1B). The side-by-side orientation is obtained from the crystal lattice of the published structure and has been inferred as the potential dimer by our program. In this model, the C-termini of two monomers are separated by a shorter distance of 42.8 Å. A corresponding C-terminal domain that matches, has an N-terminal separation of 41.7 Å (Bashir *et al.,* 2023).

The C-terminal domain here is organized in a side-by-side orientation. Interestingly, this orientation has been suggested to be the correct dimerization state of the IRE1 C-terminal domain (Joshi *et al* ., 2015). In our case the lumenal domain dimer (Figure 1B) is less stable form with 1130 Å of interface area, and -12.3 and 0.3 kcal/mol of theoretical interaction and dissociation free energy, respectively. In contrast, the other model depicts a more stable form of the quaternary structure of IRE1 (Figure 1A) with 1730 Å of interface area, and -15.2 and 7.2 kcal/mol of theoretical interaction and dissociation free energy, respectively. It means that in our model, the dimer has a reasonably large interface area and a low dissociation free energy indicating that it can easily undergo monomer-dimer transition. Also, an approximate 40 Å separation between the luminal---C-terminal domain tethers are more likely to have stable structure that stabilizes the full IRE1 protein in its functional form.

The activation of the catalytic activity of IRE1 requires its dimerization; however, the correct orientation of dimerization needed for activity is ambiguous because the cytoplasmic domain of protein showed head-tail dimerization in ADP bound form and side-by-side dimerization in the apo-form. This indicates that the interface dissociation energy is very low in both the dimeric states of the protein. The head-tail arrangement has an interface area of 1093 Å, but -1.6 kcal/mol of computed interface free energy contributed by 7 hydrogen bonds and 3 salt bridges. On the other hand, the side-by-side dimer has 1151 Å2 of interface area, has interaction free energy of +8.9 kcal/mol contributed by 33 hydrogen bonds and 32 salt bridges. It is obvious from the calculations that the side-by-side dimer is stabilized by entropic forces because theoretical free energy value is positive for a complex that has been experimentally determined. It also means that the stability of this complex will be tightly coupled to the immediate environment where it exists. On the other hand, the head-tail dimer is much more stable through enthalpic contributions and will need higher activation-deactivation energy to alter its dimerization state. The side-by-side dimer is more suitable to act as a sensor protein. These results collectively suggest that our quaternary structural model presents a desired conformation of IRE1 lumenal dimer that is in synchrony with the potential side-by-side orientation of cytoplasmic dimer.

## Methods

The quaternary structures analysis of IRE1 protein ( **accessionID O75460** ) was done using IPAC (Inference of Protein Assembly in Crystals). This program detects quaternary structures from crystal lattice by utilizing a scheme where point group symmetry works in combination with naïve Bayes classifier under Boolean framework (Mitra *et al.,* 2011). In the first step, by utilizing the asymmetric unit information available from the PDB file, we generate all symmetry- related molecules in the lattice. Non-protein atoms are also included. In this step, we also check presence of disulfide bonds at the interface, which are grouped together as functional units (FUs). In the next step, biological interfaces are detected by applying Bayes classifier. Smaller interfaces are connected into a large single interface. A depth-first search is used in a cyclic manner until no new interfaces are detected. In the last step, point group symmetry (PGS) is used to screen merged FUs. Cyclic PGS is applied to identify FUs of size n (>2). Symmetry with a threshold of 10.0 Aº can be identified by using backbone-trace Ca atoms. The largest FU satisfying PGS is inferred as the quaternary structure of the protein. In this particular study we have used an updated version of this program that emphasizes the contribution of symmetry in determining the final quaternary structure (yet to publish).
